# Establishment of an animal model of adjacent segment degeneration after interbody fusion and related experimental studies

**DOI:** 10.1186/s13018-023-04072-1

**Published:** 2023-09-07

**Authors:** Song Chen, Shiqi Suo, Zhitao Xie, Jinglan He, Jiaqi Li, Dengke Duan, Guoyong Qiao, Wei Zhang

**Affiliations:** 1 Department of Orthopaedics, The Affiliated Hospital of Hebei Engineering University, No. 81, Congtai Road, Congtai District, Handan, 056000 China; 2 Department of Gynecology, The Affiliated Hospital of Hebei Engineering University, Handan, 056000 China; 3 Department of Spine Surgery, The Third Hospital of Hebei Medical University, No. 139 Zi-Qiang Road, Shijiazhuang, 050000 Hebei China; 4 Department of Orthopaedics, Handan First Hospital, Handan, 056000 China

**Keywords:** Adjacent segment degeneration, Animal model, Tumor necrosis factor-α, Interleukin-1β

## Abstract

**Background:**

Degenerative spine conditions are common and frequent clinical diseases, and adjacent segment disease (ASD) after spinal fusion (SF) is a common complication after spinal fusion (SF). In this study, we established an animal model of ASD after interbody fusion to observe the morphologic changes of adjacent segment (AS) disks and to determine the expression and significance of tumor necrosis factor-alpha (TNF-α) and interleukin-1beta (IL-1β) in ASD tissues to provide a good experimental basis and reference for clinical prevention and treatment of ASD after interbody fusion.

**Methods:**

Thirty-six male and female New Zealand rabbits weighing 2.0–2.5 kg were randomly divided into control group (group A) and experimental groups (groups B, C, and D), with 9 rabbits in each group, of which groups B, C, and D were the 4-, 8-, and 12-week groups, respectively. Autologous iliac bone grafts were used as the bone graft material. In the experimental groups, a SF was performed on the C2–C3 intervertebral space. The C3–4 adjacent segments were examined. In the experimental group, the animals were subjected to gross observation, X-ray examination, hand touch inspection, and micro-computed tomography (micro-CT) 4, 8, and 12 weeks after surgery. The micromorphologic changes of the cervical disks in the segments of the control group and experimental groups were observed under light microscopy. Immunohistochemistry and Western blotting were used to detect the expression of TNF-α and IL-1β in the AS tissues after interbody fusion in the control and experimental groups.

**Results:**

The measurement data of the rabbit cervical spine bony structures indicated that the length of the vertebral body and the sagittal diameter of the lower end of the vertebral body decreased gradually from the 2nd–6th cervical vertebrae, and the difference was statistically significant (*P* < 0.05). The difference in the transverse diameter of the lower end of the vertebral body was not statistically significant (*P* > 0.05), the change in the oblique diameter of the lower end of the vertebral body fluctuated, and the difference was statistically significant (*P* < 0.05). The fusion rate of the cervical spine by hand touch inspection was 22.2% (2/9), 55.6% (5/9), and 88.9% (8/9) in groups B, C, and D, respectively. The differences in bone volume-to-total volume (BV/TV) and X-ray scores were statistically significant in groups B, C, and D (*P* < 0.05). Significant degeneration occurred in groups B, C, and D compared with group A. The expression of TNF-α and IL-1β in the intervertebral disk tissue was significantly higher in groups B, C, and D compared with group A (*P* < 0.05), and increased with time.

**Conclusion:**

In this study, an animal model of ASD after interbody fusion fixation in rabbits was successfully established. Postoperative imaging and hand touch inspection showed a positive correlation between the amount of new intervertebral bone and the degree of fusion with time. The results of immunohistochemistry and Western blot showed that TNF-α and IL-1β were highly expressed in the AS tissues of the experimental group after interbody fusion, and the degree of disk degeneration was positively correlated with the time after interbody fusion.

## Background

Spinal fusion (SF) is a classic surgical procedure for the treatment of degenerative spinal disorders, but the rigid fixation of the internal fixation results in the loss of fusion segment motion and an altered biomechanical environment of the spine. In recent years, the problem of adjacent segment disease (ASD) caused by SF has become increasingly prominent [1–3] and a common complication after SF [4]. ASD refers to the degenerative changes in the upper or lower segment adjacent to the fusion segment, including disk herniation or height loss, osteophyte formation, process hyperplasia, and spondylolisthesis. Understanding the surgical risk factors associated with the emergence of ASD is crucial for prevention because ASD is a major contributor to disability. The development of ASD has been significantly influenced by various surgical techniques, including decompression without fusion, non-instrumented fusion, and instrumented fusion [5]. How to overcome the shortcomings of rigid fixation with pedicle screws, maintain the stability of the spine while preserving the physiologic function of a fused segment part, and reduce the incidence of ASD has become a major focus of research interest [3, 6]. In recent years, new techniques and methods, such as non-fusion fixation of the spine [7, 8], minimally invasive endoscopic surgery of the spine [9, 10], and artificial disk replacement, have been introduced [11–13].

The mechanism underlying ASD after SF has not been established, and most studies on ASD after SF are clinical case follow-up reports and cadaveric model biomechanical experiments [14, 15]; however, the clinical studies have been mostly retrospective with a lack of prospective studies. Although cadaveric model biomechanical experiments simulate the internal fixation of spinal interbody fusion, cadaveric model biomechanical experiments cannot simulate the process of interbody fusion and late ASD, which lacks convincing power. Many studies have been conducted domestically and internationally to clarify the pathogenesis and influencing factors, but the different research methods and sample sizes, as well as the complexity of the influencing factors, have led to different and controversial findings. Most studies have suggested that biomechanical changes, such as increased mobility of adjacent segments and increased pressure load on the intervertebral disks and synovial joints after spinal interbody fusion are important mechanisms underlying ASD [16]. Some studies, however, have suggested that ASD after SF may be a natural degenerative process with aging and not related to interbody fusion [17]. It has been reported that factors, such as age [18], gender [19], weight [20], osteoporosis [21], pedicle screw placement [22], sagittal balance, and posterior vertebral complex disruption [23], may increase the incidence and risk of ASD, but the correlation between each influencing factor and ASD remains somewhat controversial. In view of the above findings, the current study effectively controlled the non-experimental and experimental factors by establishing a good animal model of SF to reduce the bias of the study results and simulated the occurrence, development, and regression process of ASD after SF in the clinical setting to the greatest extent possible. To provide a good experimental basis and reference for clinical prevention and treatment of ASD after SF, we established an animal model of ASD after SF to observe the morphologic changes of adjacent segment (AS) disks and determined the expression and significance of tumor necrosis factor-alpha (TNF-α) and interleukin-1beta (IL-1β) in ASD tissues.

## Materials and methods

### Experimental materials

#### Experimental animals

Thirty-six New Zealand White rabbits of either gender, 16–24 weeks old and weighing 2.0–2.5 kg, were provided by the Experimental Animal Center of the Third Hospital of Hebei Medical University (SCXK [JI] 2016-002-181104). The animals were randomly divided into a control group (group A) and experimental groups (groups B, C, and D), with 9 animals in each group. In the control group (group A), the anatomic structure of the rabbit cervical vertebrae was measured and preserved for examination, no experiments were done on these animals. In the experimental groups, a SF was performed on the C2–C3 intervertebral space, and autologous bone grafts harvested from the iliac crest were used as bone grafting materials. The C3–4 adjacent segments were examined. Groups B, C, and D were the 4-, 8-, and 12-week groups, respectively. The study complied with the animal ethics requirements and was approved by the Ethics Committee of our institution.

### Animal model establishment

#### Preoperative preparation and anesthesia

The rabbits were housed in single cages for 1 week before surgery, fasted for 6 h before surgery, weighed accurately and recorded, and general anesthesia was performed by slow injection of 20% urethane (3.5 mL/kg, BOSF, W001, BASF SE China, Hefei, China) through the ear marginal vein. Surgical instruments and built-in objects were routinely autoclaved.

#### Surgical method

Experimental groups: After successful anesthesia, the rabbit was placed on the operating table in the lateral position. Autologous bone harvested from the iliac crest was routinely disinfected, then a surgical incision approximately 2 cm in length was made, the subcutaneous soft tissue was exposed layer-by-layer, bluntly separated to the ala of the ilium, and a granulated bone graft approximately 3 × 4 mm was removed with a small bone chisel. The surgical field was rinsed with saline to stop bleeding, then sutured layer-by-layer and bandaged. After the iliac bone was extracted, the rabbit was placed in the supine position and after disinfection with iodine, a surgical incision of approximately 4 cm in length was made in the anteromedial part of the right cervical spine. The 2nd–3rd cervical vertebral space was located during surgery, and blunt dissection of the longus colli muscle, carotid sheath, and tracheoesophageal nerve sheath was carefully performed, then peeled away layer-by-layer until the anterior cervical vertebral body was reached.

The cervical 2nd–3rd vertebral space and upper and lower vertebral bodies were exposed using a homemade periosteal stripper. The cervical 2nd–3rd intervertebral disk and endplate were removed with a homemade reamer, and the autogenous iliac bone of the experimental rabbit was removed in advance to be used as the interbody fusion graft placement into the cervical 2nd–3rd vertebral space. The homemade cervical spine plates (4 mm*11 mm; upper vertebral body adjacent to the lower endplate and lower vertebral body adjacent to the upper endplate) were placed and drilled and homemade screws with a diameter of 1.7 mm and a length of 5 mm were placed. The surgical area was repeatedly rinsed with saline to stop bleeding, then wrapped with sutures layer-by-layer, and the molding was completed. No intervention was performed in the control group and the anatomic structure of the rabbit cervical vertebrae was measured and preserved for examination.

#### Postoperative treatment

The rabbits were reared separately in cages postoperatively and routine dressing changes at the incision site were performed daily. Penicillin (50,000 U, CSPC Pharmaceutical Group Limited, Shijiazhuang, China) was injected intramuscularly for the first 3 days postoperatively. The experimental animals were disposed of by sealed incineration after sampling and death.

### Observation methods and indices

#### Gross observation

The experimental animals were observed for postoperative feeding, activity, and response to external stimuli. The incidence of surgical complications, including death, neurovascular injury, dural sac tear, postoperative incision infection, loose displacement of internal fixation, and spinal cord injury, was determined.

#### Imaging examination

Cervical spine anterior–posterior and lateral view X-ray examinations were performed 4, 8, and 12 weeks postoperatively in groups B, C, and D. The position of the plates and screws and interbody fusion in each group was observed, and whether there was any displacement or loosening of the built-in material was determined. After the examination, the animals were sacrificed by air embolization, and the cervical specimens of the fused segments were removed for hand touch inspection by two physicians using a double-blind method (Cohen’s Kappa = 0.907). The soft tissues and internal fixations (homemade plates and screws) around the fused segment were removed, and only the bony part of the vertebral body was preserved, then flexion and extension (flexion and dorsiflexion; bend forward and stretch back), lateral bending (bend the neck to one side), and torsion (side-to-side rotation) were performed to detect the presence of activity of the fused segment. The judgment criteria were as follows: the fused segment inactivity shows fusion (yes); or the segment activity indicates non-fusion (no).

Then, the cervical specimens with the soft tissue stripped from the fused segment were examined using micro-CT (SkyScan 1173 [18 μm resolution; Pixels, 2240 × 2240] Bruker, Belgium;) for each group of specimens to assess intervertebral neonatal bone production and bony fusion in the fused segment. The bone volume-to-total volume (BV/TV) of the neonatal bone volume and implant material in groups B, C, and D were determined using the analysis software(version 1. 1 [build 12], comes with SkyScan 1173). Readings were performed by two physicians using a double-blind method to determine interbody fusion in the fused segment and scored according to the presence or absence of bone formation or fusion (Cohen's Kappa = 0.912). Table 1 shows the grading definition of the radiographic grading system of the micro-CT.

**Table 1:** Radiographic grading system

Items	Scores
No bone formation	0
Small amount of new bone formation and definite pseudo-joint	1
Moderate bone formation and definite pseudo-joint	2
Good new bone formation and possible pseudo-joints	3
Good new bone formation and possible pseudo-joint	4
Definite fusion	5

The X-ray source, experimental sample, and detector were placed in sequence along the direction of the measurement optical axis, thus the experimental sample was located between the X-ray source and detector. The positions of the X-ray source, experimental sample, and detector along the optical axis were kept unchanged, and the experimental sample was perpendicular to the measurement optical axis. The two X-ray projection images of the experimental sample were obtained at the current angle and after a 180° rotation. The dimensions of the same feature structure in the experimental sample were measured separately in the two above-mentioned X-ray projection images, and the real dimensions of the feature structure were calculated.

#### HE staining observation

After sacrificing the experimental animals, the disk tissues of the adjacent segments (3 and 4 cervical vertebrae) were removed from each group after fusion, then fixed in formaldehyde solution. After dewaxing and hydration, the tissues were paraffin-embedded (JB-P5, WuHanJunJie Electronics Co., LTD, Wuhan, China) and sectioned (RM2016, Shanghai Leica Instrument Co., LTD, Shanghai, China). HE staining was then performed as follows: the tissues were first stained (Giotto, DIAPATH, Italy) with hematoxylin dye solution for 5 min, washed, differentiated, washed, blued, and rinsed; the slices were dehydrated (Donatello, DIAPATH, Italy) and eosin dye was added for 5 min; the slices were dehydrated and sealed with neutral gum; and the slices were placed under the light microscope (Eclipse E100, Nikon, Tokyo, Japan) to observe the morphologic structural changes of the nucleus pulposus and fibrous ring tissue.

#### Immunohistochemical observation

After the experimental animals were sacrificed, the disk tissues of the adjacent segments (3rd–4th cervical vertebrae below the fusion site of the 2nd–3rd cervical vertebrae) were removed after fusion in groups B, C, and D and the disk tissues of the same segments (3rd–4th cervical vertebrae below the fusion site of the 2nd–3rd cervical vertebrae) in group A were subjected to immunohistochemistry to detect the expression of TNF-α and IL-1β.

Examination standard: The intensity of cell positivity (antigen content) was quantified as follows: weak positive ( +),1 point; medium positive (+ +), 2 points; and strong positive (+ + +), 3 points. The number of positive cells was quantified as follows: weakly positive (total number of + positive cells < 25%); moderately positive (total number of +  + positive cells between 25 and 49%); and strong positive (total number of +  +  + positive cells > 50%). The calculation formula was as follows: ( +)% × 1 + (+ +)% × 2 + (+ + +)% × 3. The total value < 1.0 was ( +), 1.0–1.5 was (+ +), and > 1.5 was (+ + +). At least 5–10 high-power fields were observed at random.

#### Western blotting

The levels of TNF-α and IL-1β protein expression in the disk tissues of adjacent segments (3rd–4th cervical vertebrae below the fusion site of 2nd–3rd cervical vertebrae) in groups B, C, and D after SF and in the disk tissues of the same segments (3rd–4th cervical vertebrae below the fusion site of 2nd–3rd cervical vertebrae) in group A were quantified by protein blotting. The intervertebral disk tissue was washed and cut with phosphate buffered saline, homogenized with homogenate beads, then the intervertebral disk tissue homogenate was prepared with the lysate. The supernatant was collected after centrifugation at 12,000 rpm at 4 °C for 10 min. The protein concentration was measured with the BCA protein concentration determination kit (company, city, state, country), according to the kit instructions. The reducing protein loading buffer was added and the supernatant was stored in a refrigerator at − 20 °C. The protein samples were isolated by SDS-PAGE electrophoresis and transferred to PVDF membranes (0.45 µm). The filter membrane was closed with skim milk powder at room temperature for 30 min, and the primary antibody was added and incubated in a shaker overnight at 4℃. Then, the secondary antibody was added and incubated for 30 min, and the color developing agent was added after washing the film. Chemiluminescence and gel image analysis (ChemiScope 6300, CLINX, Shanghai, China) were performed on the films.

### Statistical methods

The statistical analysis was performed using SPSS 25.0 (IBM Corp., Armonk, NY, USA). To compare the variations across groups, a one-way ANOVA-SNK-q test was used. Statistics were deemed significant at a *p* < 0.05.

## Results

### Anatomic structure of rabbit cervical vertebrae and plate screw specifications (Group A)

**Fig. 1:**
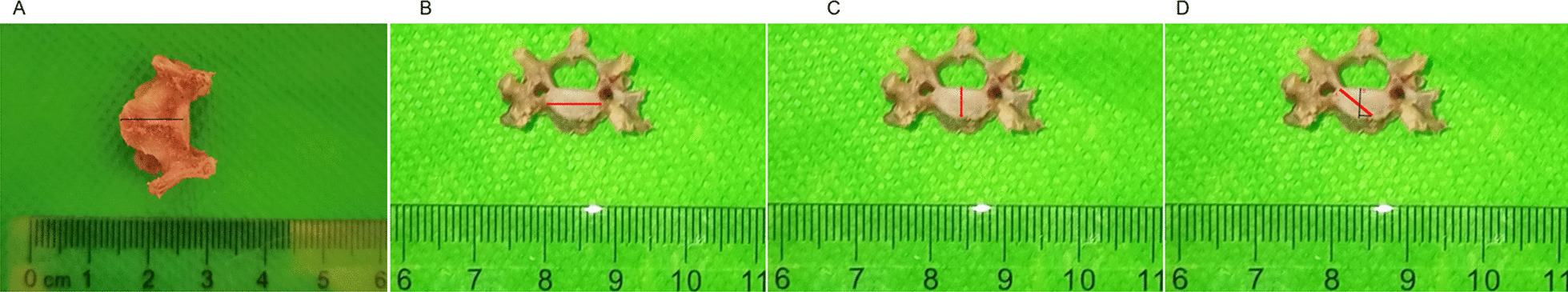
Measurement of bony structures of the rabbit cervical spine. Vertebral body length (**A**), vertebral body transverse diameter (**B**), vertebral body sagittal diameter (**C**), vertebral body oblique diameter (**D**)

**Fig. 2:**
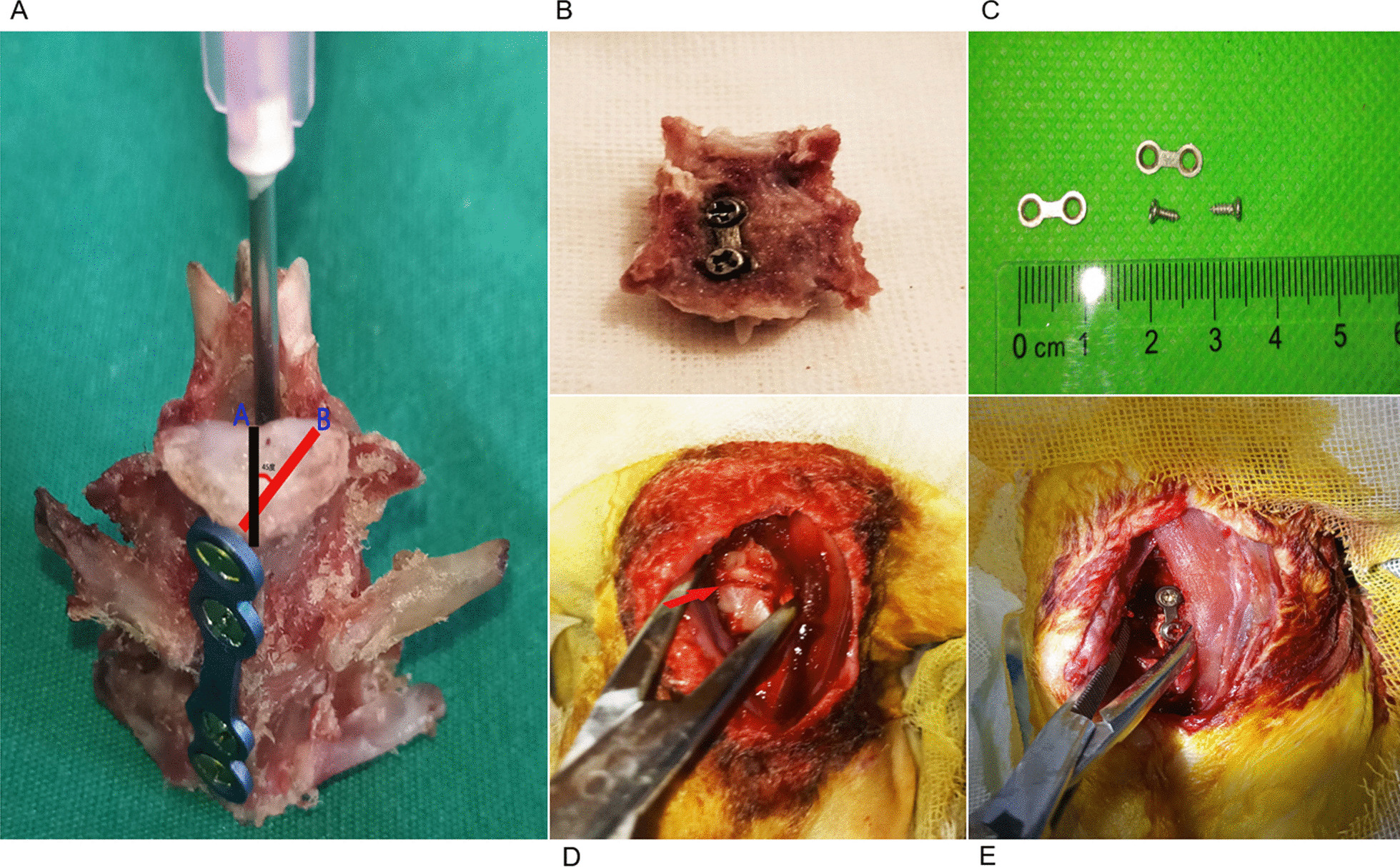
Schematic diagram of internal fixation device placement. Line A represented the sagittal diameter; line B represented the oblique diameter, the angle between lines A and B was 45°, screw implantation and oblique diameter direction avoids entering the spinal canal and damaging the spinal cord (**A**), the vertebral body was placed into the internal fixation, and the internal fixation was attached to the bone surface (**B**), the plate and screw specification schematic (**C**), the red arrow shows the vertebral space (**D**), the plate and screw were placed (**E**)

The cervical vertebrae of rabbits are thick at both ends and slightly thin in the middle, with a thin waist drum shape. The anterior ridge of the vertebral body is elevated, and the sides of the vertebral body are relatively flat. The sagittal, transverse, and oblique diameters of the rabbit cervical vertebrae and the vertebral body length measurements are shown in Table 2 and Fig. 1. The oblique diameter is defined by a 45° angle between the highest part of the bone crest, and is 1 mm from the sagittal diameter. The differences were statistically significant (*P* < 0.05) from the 2nd–6th cervical vertebrae body lengths and the sagittal diameter of the lower vertebral body, while the differences were not statistically significant (*P* > 0.05) from the 2nd–6th cervical vertebral body lower transverse diameters and statistically significant (*P* < 0.05) from the 2nd–6th cervical vertebral body lower oblique diameters (Table 2). Based on the anatomic data of the rabbit cervical vertebrae, screws with a diameter of 1.7 mm and a length of 4 mm, and plates with a length of 11 mm and a width of 4 mm were prepared. The plates were placed laterally and anteriorly to the vertebral body for fixation, and the screws were placed in a direction parallel to the oblique diameter of the vertebral body (Fig. 2).

**Table 2:** Measurement data of cervical vertebra body in rabbits (control group [group A], n = 9, mean ± SD, mm)

Vertebral sequence	Vertebral body length	Sagittal diameter of the lower end of vertebral body	Transverse diameter of the lower end of vertebral body	Oblique diameter of the lower end of vertebral body
C2	11.92 ± 0.44	3.91 ± 0.51	7.20 ± 0.98	4.79 ± 0.45
C3	10.12 ± 0.58^*^	3.76 ± 0.33	6.84 ± 0.73	4.46 ± 0.29^*^
C4	9.79 ± 1.03^*^	3.61 ± 0.27^*^	6.79 ± 0.86	4.96 ± 0.36^#^
C5	8.54 ± 0.98^*#△^	3.42 ± 0.21^*^	6.37 ± 0.65	4.60 ± 0.23
C6	7.39 ± 0.95^*#△☆^	3.32 ± 0.13^*#^	6.23 ± 0.42	4.26 ± 0.21^*^
*F* value	38.060	6.893	2.410	6.575
*P* value	< 0.001	< 0.001	0.065	< 0.001

Compared with C2, *P < 0.05; compared with C3, ^#^*P* < 0.05; compared with C4, ^△^*P* < 0.05; compared with C5, ^☆^*P* < 0.05 (SNK-Q test)

### Gross observation

On the day after operation, the diet, activity levels, and response to external stimuli of the experimental animals were poor. On the first postoperative day, the response to external stimuli was restored, but the diet and activity levels were still poor. On the second postoperative day, the diet, activity, and response to external stimuli had returned to normal. In the early stage of the experiment, three animals died due to an anesthetic overdose or excessive speed, including one in group B and two in group D. One animal died due to a postoperative infection in group C.

### Imaging observation

**Fig. 3:**
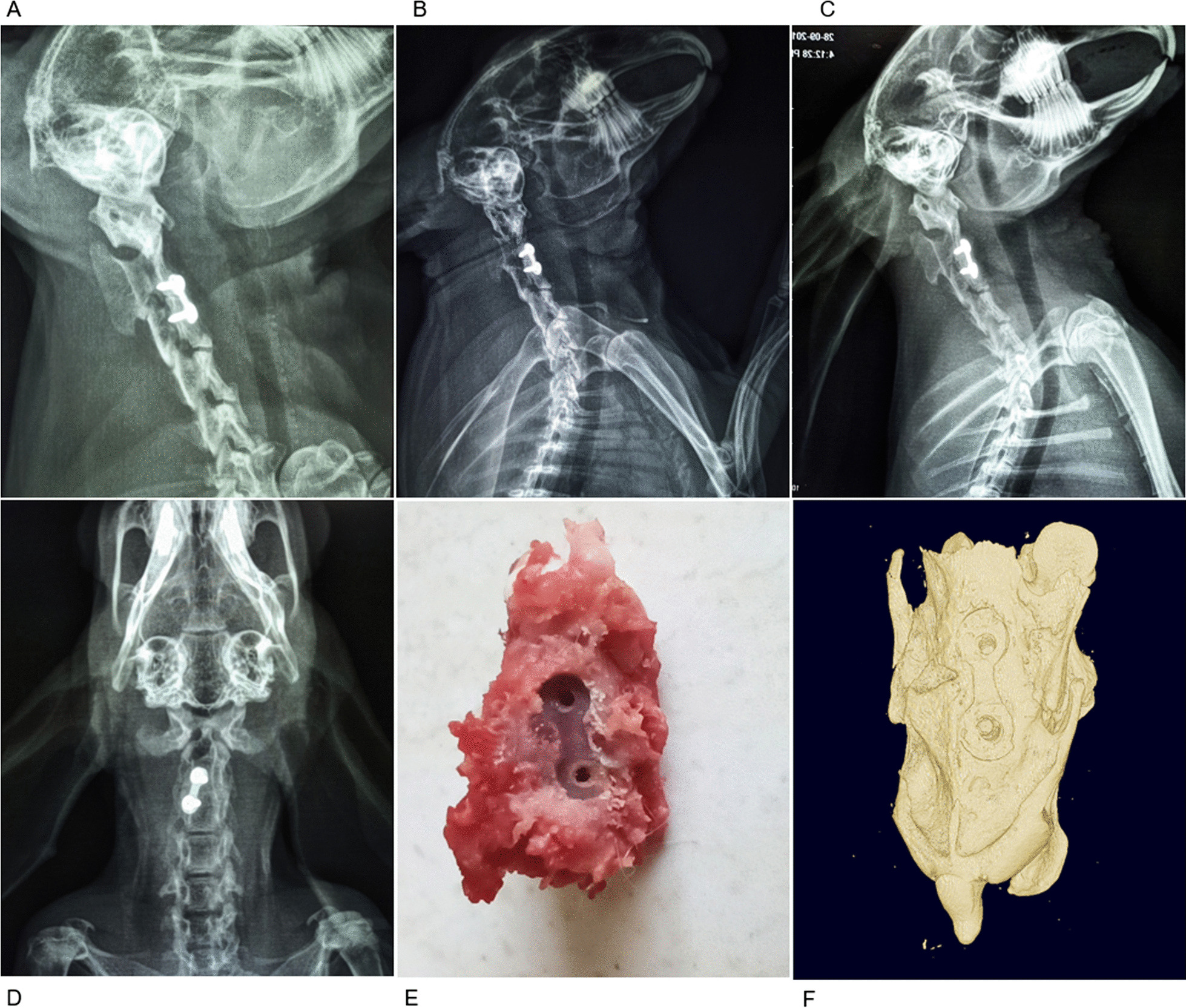
Gross observation of postoperative cervical spine radiographs and fused segments in the experimental group. Lateral cervical spine radiographs 4 (**A**), 8 (**B**), and 12 (**C**) weeks postoperatively, orthogonal cervical spine radiographs showing the placement of the internal fixation in the lateral anterior part of the cervical spine (**D**), general view of the fused segment with the internal fixation removed (**E**), and micro-CT three-dimensional reconstruction of the fused segment (**F**)

The cervical spine X-ray anterior–posterior and lateral views revealed that the internal fixation position was good in group B. An obvious vertebral space was visible, among which a screw was loosened and retracted in one animal; the other animals had no internal fixation fractures or displacement. The internal fixation position was good in groups C and D, and there was no internal fixation loosening or fractures. The vertebral space in group C was blurred, and bone scab growth was visible. The vertebral space in group D was connected by bone with the upper and lower vertebral bodies (Fig. 3).

Micro-CT results showed that the intervertebral space was still clear in group B, despite a small amount of bone crust formation. There was a large amount of bone crust growth and the intervertebral space was blurred in group C. There was a continuous bony connection visible in the intervertebral space in group D, indicating that interbody fusion had occurred. A statistically significant difference existed between the BV/TV ratio and X-ray scores (*P* < 0.05; Table 3).

**Table 3:** Micro-CT detection of bone volume fraction and radiographic score at weeks 4, 8, and 12 (mean ± SD)

Groups	BV/TV(%)	Micro-CT points
B	32.73 ± 4.97	1.89 ± 0.78
C	53.50 ± 7.23^*^	3.22 ± 0.67^*^
D	69.91 ± 4.88^*#^	4.44 ± 0.52^*#^
*F* value	93.000	33.080
*P* value	< 0.001	< 0.001

BV:BT: bone volume-to-total volume ratio

Compared with group B, *P < 0.05; compared with group C, ^#^*P* < 0.05 (SNK-Q test)

### Hand touch inspection results

Interbody fusion occurred in 2 animals in group B, with a fusion rate of 22.2% (2/9), 5 animals in group C, with a fusion rate of 55.6% (5/9), and 8 animals in group D, with a fusion rate of 88.9% (8/9).

### HE staining

**Fig. 4:**
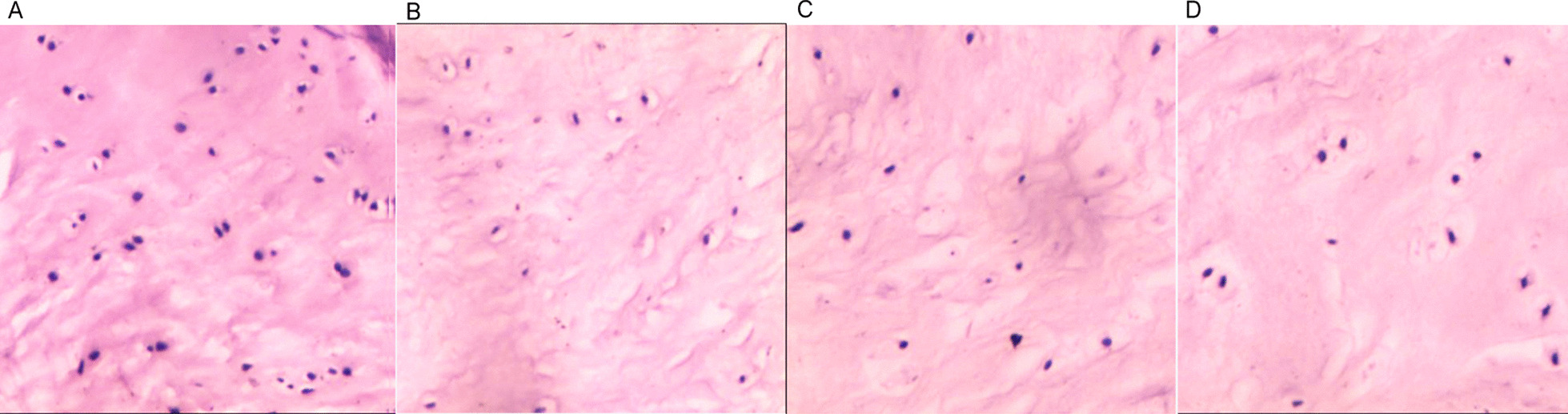
Hematoxylin–eosin staining (HE staining) results (200×). HE staining results of disk tissue in the control group (**A**), 4 weeks postoperatively (**B**), 8 weeks postoperatively (**C**), and 12 weeks postoperatively (**D**), the degree of degeneration was positively correlated with the postoperative time

The intervertebral disks in groups B, C, and D showed structural disorders between the fibrous ring and the nucleus pulposus, with collagen fibrous hyperplasia, reduced nucleus pulposus cells, and different degrees of nucleus pulposus wrinkling and gaps, which became more apparent with a prolongation of postoperative time (Fig. 4).

### Immunohistochemistry

**Fig. 5:**
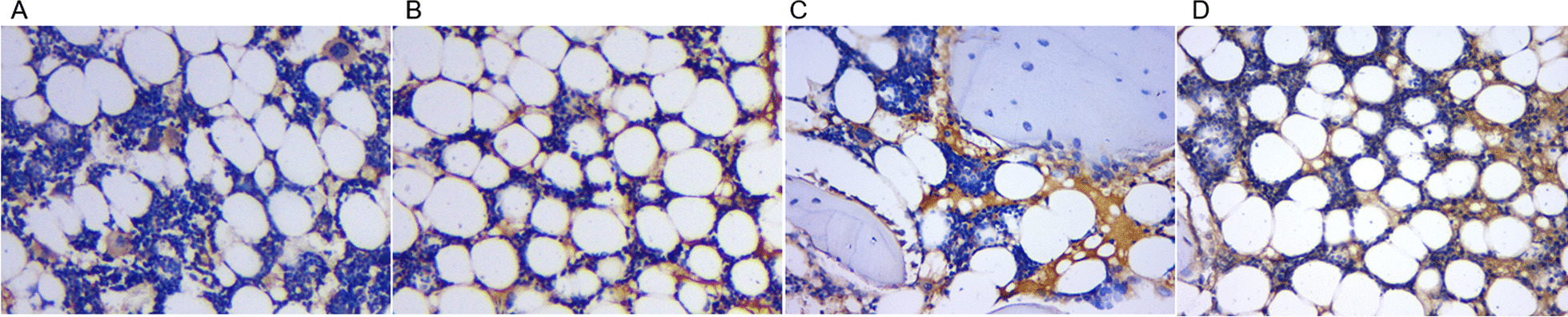
IL-1β immunohistochemical results (400×). There was less positive IL-1β staining in the cervical disk tissue of the control group (**A**), and positive IL-1β staining in the cervical disk tissue 4 weeks postoperatively (**B**), 8 weeks postoperatively (**C**), and 12 weeks postoperatively (**D**) gradually increased the number of positive cells and intensified the staining with the postoperative time

**Fig. 6:**
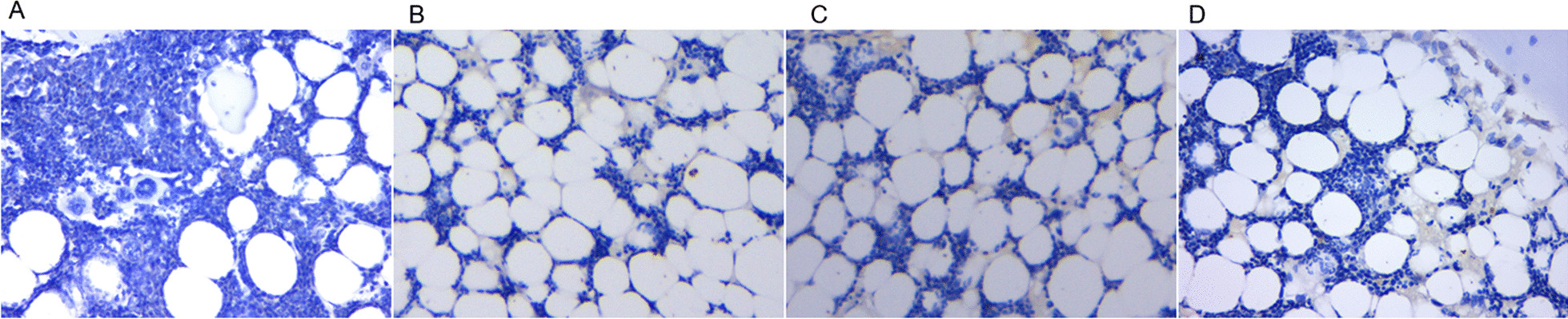
Immunohistochemical results of TNF-α (400×). The number of positive TNF-α staining in cervical disk tissues in the control group was less (**A**), and the number of positive cells in cervical disk tissues 4 weeks (**B**), 8 weeks (**C**), and 12 weeks (**D**) after surgery gradually increased and the staining gradually intensified with prolongation of the postoperative time

The immunohistochemical results showed positive IL-1β and TNF-α protein expression (brown-yellow or light brown-yellow) and the hematoxylin-stained nuclei were blue. The positive TNF-α and IL-1β immunohistochemical staining in the group A disk tissues had decreased expression. The number of positive cells gradually increased with time and the staining gradually deepened with time (Figs. 5 and 6).

### Western blotting

**Fig. 7:**
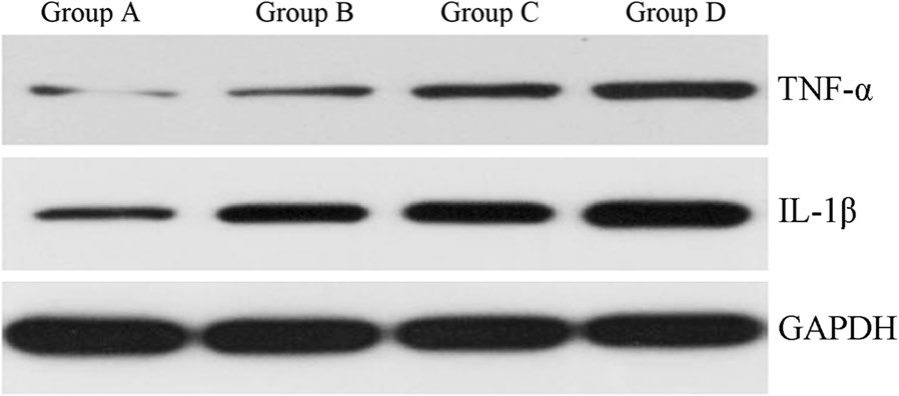
Western blotting for TNF-α and IL-1β. The control group AS tissue expressed weaker TNF-α and IL-1β proteins, and the rabbits gave interbody fusion molded AS tissue showed a significant increase in TNF-α and IL-1β protein expression, both of which were significantly different compared with the control group (*P* < 0.05)

TNF-α and IL-1β proteins were weakly expressed in the group A cervical disk tissues, while TNF-α and IL-1β protein expression in the AS tissues of groups B, C, and D after interbody fusion increased considerably, and the difference was statistically significant compared with group A (*P* < 0.05, Fig. 7 and Table 4), and the increase was more and more apparent with time.

**Table 4:** Comparison of TNF-α and IL-1β protein expression between groups (mean ± SD)

Groups	TNF-α	IL-1β
A	0.36 ± 0.032^*∆#&^	1.32 ± 0.022^*∆#&^
B	0.68 ± 0.019^*#&^	2.11 ± 0.029^*#&^
C	1.04 ± 0.027^*∆&^	2.43 ± 0.033^*∆&^
D	1.85 ± 0.034^*∆#^	2.98 ± 0.021^*∆#^
*F* value	4531.65	5927.72
*P* value	< 0.001	< 0.001

^*^Compared with control group, *P* < 0.05; ^∆^compared with 4 weeks after operation, *P* < 0.05; ^#^compared with 8 weeks after operation, *P* < 0.05; ^&^compared with 12 weeks after operation, *P* < 0.05

## Discussion

In the selection of animals to establish an ideal animal model for SF, the selected animals need to have a wide, parallel endplate capable of performing interbody fusion operations. Primates are undoubtedly the best choice for such a model [24], but are limited by ethical and sample size restrictions. Large animals, such as sheep [25], dogs [26], and pigs [27], are susceptible to space, financial, and size limitations due to complex perioperative management, which affects the true reliability of the study results. Although small animals are easy to keep and manage and are not restricted in terms of space, funding, and size, it is difficult to observe the degree of interbody fusion after SF because the vertebral bodies are smaller and not favorable for fixation. Compared to large animals, such as goats, dogs, and pigs, rabbits are appropriately priced, can be maintained in large numbers, are easy to manage, and yield reproducible results. Compared with rats or mice [28], rabbits are appropriately-sized, have a wider endplate, are easy to operate on, and are more tolerant of surgery. Valdes et al. [29] and Palumbo et al. [30] reported an animal model involving posterior lateral rabbit lumbar intertransverse process implant fusion, but this model had a low fusion rate and did not truly mimic the mainstream interbody fusion that exists in clinical practice. The animal model of interbody fusion internal fixation is used less often by researchers because of complicated surgical operations and displacement of the bone graft, but the interbody fusion model compensates for the defects of intertransverse process bone graft fusion, which can simulate the clinical surgical approach to the maximum extent and obtain more reliable and realistic research data. There is a lack of successful small- and medium-sized animal models that can simulate clinical interbody fusion internal fixation of the spine in domestic and international studies.

In this study, we first performed anatomic measurements related to the cervical vertebrae of the rabbit and determined the specifications of internal fixation after obtaining anatomic data. To ensure the safety and stability of the inserted plates and screws, we chose to fix the plates on the lateral anterior aspect of the rabbit cervical spine. This area is relatively flat, which facilitates the attachment and fixation of the plate to the vertebral body. The sagittal, transverse, and oblique diameters of the vertebral body at the lower end of the vertebral body 2 mm from the end plate are larger, and screws are inserted in the same direction as the oblique diameter of the vertebral body, which reduces the risk of injury to the spinal cord due to screws entering the spinal canal.

Our results showed that there was one case of postoperative paralysis involving both lower limbs in group C due to a possible intraoperative injury to the spinal cord, but the remaining animals did not have postoperative paralysis due to contusion of the spinal cord by screws entering the spinal canal. One case of screw loosening and retraction occurred in group B, but the remaining animals showed good positioning of internal fixation and no internal fixation loosening or fractures, indicating that the internal fixation method used in these experiments was safe and effective. Three animals died in the early stage of modeling due to over-injection or over-rapid injection of an anesthetic. After adjusting the dose of anesthetic and improving the injection technique, no experimental animal deaths due to anesthesia occurred again in the middle and late stages of the experiment. When injecting urethane, one-third of the total dose should be delivered quickly and the remaining two-thirds of the dose should be delivered slowly and uniformly according to the principle of “first fast and then slow.”

One postoperative infection death in this study may have been caused by contamination of surgical instruments or a postoperative incision infection. One death in group C was caused by spinal cord injury due to inadvertent intraoperative manipulation, resulting in bilateral lower limb paralysis. This death resulted in increased gentle and careful handling of disk tissue and endplates. In addition, according to the anatomic measurements, the cervical intervertebral space of rabbits had a specific angle, which was slightly tilted upward and posteriorly.

In this study, micro-CT was used to detect and evaluate neonatal bone production and interbody fusion. Micro-CT is the most widely used computerized body imaging system with which bone metabolism is studied to evaluate bone morphology and microarchitecture [31]. Micro-CT has high resolution and ease of use and can obtain information on the internal three-dimensional structure of the examined bone tissue.

The BV/TV ratio reflects the bone volume of bone trabeculae in different samples. An increase in the BV/TV ratio indicates that bone anabolism is greater than catabolism and bone volume increases [31]. The BV/TV ratio showed that bone volume increased as follows: group D > group C > group B. The difference in the BV/TV ratio between groups B, C, and D was statistically significant (*P* < 0.05). The combination of X-ray, micro-CT, and hand touch inspection showed that the degree of interbody fusion and neonatal bone volume was positively correlated with the postoperative time.

In this study, the experimental group rabbits were sacrificed 4, 8, and 12 weeks postoperatively. The animals were then taken for examination, in which HE staining showed disorganized disk tissue structure, proliferation of collagen fibers, reduction of nucleus pulposus cells, and different degrees of wrinkling of the nucleus pulposus. Compared with the control group, the histopathologic changes in the intervertebral fixed fusion AS were apparent in the experimental group and gradually increased with time. The immunohistochemical results showed that most of the intervertebral disk tissues in the experimental group had positive IL-1β and TNF-α expression on immunohistochemical staining, and the number of positive cells gradually increased and the staining gradually intensified with time. These results suggested that the inflammatory cytokines, TNF-α and IL-1β, play an important role in the progression of adjacent segment disk degeneration. The main pathologic processes of immunodeficiency disease include the production of pro-inflammatory mediators, progressive loss of extracellular matrix, cell aging and autophagy, changes in the intervertebral disk cell phenotype, and a decrease in the number of active cells [32, 33]. Inflammation is a key factor in the process of disk degeneration [34]. IL-1β and TNF-α are mainly produced and secreted by immune cells, the expression of which is positively correlated with age and degree of intervertebral disk degeneration [35], and consistent with our study results.

Several studies have shown that individuals treated with SF have an increased risk of ASD [36]. Age, gender, obesity, previous degeneration, and hereditary characteristics all raise the probability of ASD. Sagittal alignment, in contrast, is important in the development of this disease [37]. Mean age, body mass index, cigarette smoking, blood pressure history, long segment fusion, high lumbosacral joint angle, pre- and postoperative L1-S1 sagittal vertical axis, postoperative lumbar lordosis, and preoperative pelvic incidence are all associated with the development of ASD according to a meta-analysis [38].

Results from Western blotting revealed that there was a significant difference between the experimental and control groups with respect to the expression of TNF-α and IL-1β protein in the intervertebral disk tissues (*P* < 0.05). These findings suggested that TNF-α and IL-1, two inflammatory cytokines, are essential in the emergence of ASD. Immune cells are primarily responsible for the production and secretion of IL-1 and TNF-α. Age and severity of ASD are strongly associated with the level of cellular expression [35], which is consistent with the experimental findings of the current investigation.

As an important member of the IL-1 family, IL-1β is involved in several disorders associated with inflammation. In fact, IL-1β produces a wide range of pro-inflammatory mediators, including cytokines and chemokines [39, 40]. IL-1β also accelerates cellular senescence [41, 42], exacerbates oxidative stress [43], and promotes angiogenesis [44]. TNF-α is a pleiotropic pro-inflammatory cytokine. TNF-α and IL-1β have an important role in ASD after SF by regulating the inflammatory response, autophagy, ECM degradation, and oxidative stress, and may be involved in the occurrence and development of ASD, but whether TNF-α and IL-1β production is a cause or a consequence of ASD and the specific mechanisms by which ASD is affected are not fully understood and warrant further in-depth study.

### Study limitations

In general, the degeneration of adjacent upper segments is more obvious, which has been confirmed by published scientific research articles. This report only observed the degeneration of adjacent lower segments, which is a limitation of this study. Moreover, the radiologic studies were not done to decide the stabilities of the operated segments after flexion and extension, lateral bending, and torsion stress test using hands. This should be further explored in further studies. Thirdly, our study, was limited by the small sample size. Fourth, another ideal animal model rather than rabbit model should be established to validate the results of this study. Thus, there is an urgent need for additional large-scale, well-designed studies focusing on ASD.

## Conclusion

In this study, an animal model of ASD after SF in rabbits was successfully established. The animal model was reasonably designed, economically efficient, simple in the modeling method, and reproducible. TNF-α and IL-1β were highly expressed in the AS tissues of the experimental groups and were positively correlated with time, suggesting that TNF-α and IL-1β have an important role in the development of ASD. The specific molecular mechanisms underlying TNF-α and IL-1β in the development of ASD are still unclear and need to be further investigated.

## Data Availability

The data related to this study can be accessed from the corresponding author.
